# Molecular and Serological Detection of *Toxoplasma gondii* in Stray Cats in Shiraz, South-central, Iran

**Published:** 2018

**Authors:** Qasem ASGARI, Iraj MOHAMMADPOUR, Razieh PIRZAD, Mohsen KALANTARI, Mohammad Hossein MOTAZEDIAN, Shahrbanou NADERI

**Affiliations:** 1. Basic Sciences in Infectious Diseases Research Center, School of Medicine, Shiraz University of Medical Sciences, Shiraz, Iran; 2. Dept. of Medical Parasitology & Mycology, School of Medicine, Shiraz University of Medical Sciences, Shiraz, Iran; 3. Research Center for Health Sciences, Mamasani Higher Education Complex for Health, Shiraz University of Medical Sciences, Shiraz, Iran

**Keywords:** *Toxoplasma gondii*, Stray cats, MAT, Nested-PCR, Iran

## Abstract

**Background:**

Toxoplasmosis is a global zoonotic disease that causes critical medical complications in neonates and immunocompromised persons. Infection rates in cats, specifically stray cats, are believed to be the best sentry of the level of *Toxoplasma gondii* in the environment. Therefore, in this study, we surveyed *T. gondii* infection in stray cats of Shiraz, one of the metropolises of Iran.

**Methods:**

The appearance of antibodies and DNA of *T. gondii* in samples from 145 stray cats was determined in order to appraise the prevalence of *T. gondii* infection, by MAT and Nested-PCR.

**Results:**

The rate of *T. gondii* infection in the cats was 69% by PCR and 82.8% by MAT. Besides, the highest rate of infection was discerned in diaphragm (37.9%) and intercostal muscle (34.5%), while the lowest rate was related to ileum (6.9%). Moreover, the similarity between MAT with titers 1:20, 1:40 and PCR were 79.2% and 86.2%, respectively (*P*=0.02 and *P*=0.0001).

**Conclusion:**

Nested-PCR and MAT are valuable techniques for molecular and serological detection of *T. gondii*. The prevalence of *T. gondii* infection in stray cats in Shiraz is high.

## Introduction

Toxoplasmosis is a globally endemic disease caused by *Toxoplasma gondii* infecting a broad spectrum of vertebrate hosts, including humans. In humans, this parasite causes rigorous medical complications such as Chorioretinitis, Lymphadenitis, Myocarditis, and Polymyositis (1). The clinical features of *Toxoplasma* infection in adults are commonly mild and includes Fever, Malaise, and Lymphadenopathy (2). Congenital Toxoplasmosis resulting in Encephalitis, Mental retardation, Microcephaly, Hydrocephaly, and Jaundice (3). Acute maternal infection can also result in abortion or neonate death (3). Among immunocompromised individuals, Toxoplasmosis is a conducting cause of hospitalization and death. In fact, restarting of dormant infection in HIV-positive patients results more prevalently in cerebral Toxoplasmosis and is a life-intimidating situation as a result of incongruous diagnosis and treatment (2, 4).

In addition, an association was found between chronic Toxoplasmosis and diseases such as Schizophrenia, Epilepsy, and other Neuropsychiatric diseases (5–8). Moreover, *T. gondii* infection was liable for substantial economic losses due to abortion in livestock animals, such as sheep and goats (9). From the other point of view, human infection is regularly obtained by consuming of tissue cysts in undercooked contaminated meat, and ingestion of oocysts from soil, water, or cat litter (10). Cats play an important role in the epidemiology of the disease, as they are the definitive hosts allowing the sexual phase of the parasite in the GI tract (11).

The feline infection can be obtained through ingestion of bradyzoites via raw meat that contains tissue cysts, and rarely oocysts; although congenital infections must also be regarded (12). The ingestion of one bradyzoite will lead to feline infection, whereas a feline must ingest 1000 oocysts to develop an infection (12). Owning just one cat increases the risk of Toxoplasmosis, but having three or more kittens makes an individual over 70 times more likely to become infected with *T. gondii* (10, 13). Infected cats shed millions of oocysts in their feces. The majority of oocysts are produced shortly after the initial acquisition of the parasite (10).

Cats infected after birth may have clinical signs, such as fever, ocular inflammation, anorexia, lethargy, hyperpnea, dyspnea, emaciation, jaundice, vomiting, diarrhea, peritoneal effusion, hypothermia, muscle hyperesthesia, and neurological abnormalities (10).

Stray cats are particularly important to public health because they are contemplated to be the best sentries of the level of *T. gondii* in the environment. This is because they wander openly without any protection from pathogens and they can get feline Toxoplasmosis by predation of infected animals with *T. gondii* cysts: birds, rodents, other wildlife, or by ingesting of undercooked meat from human waste (14).

Infection rates in stray cats give an indirect signal of the prevalence of *T. gondii* in the environment (14). Yet, little attention has been focused on this matter and there have been limited investigations of *T. gondii* infections in cats in Shiraz in recent years. Shiraz is a significant industrial and commercial metropolis in Iran. The size of the cat population has raised meaningfully with the recent improvement in people’s living standards and awareness of good animal support. In addition to microscopic examination of fecal samples, serological and molecular methods should be used as diagnostic techniques.

Therefore, in this study, we investigated *T. gondii* infection in stray cats of Shiraz, Iran, using PCR, phylogenetic analysis, and MAT.

## Materials and Methods

### Study Area

**Fig. 1:**
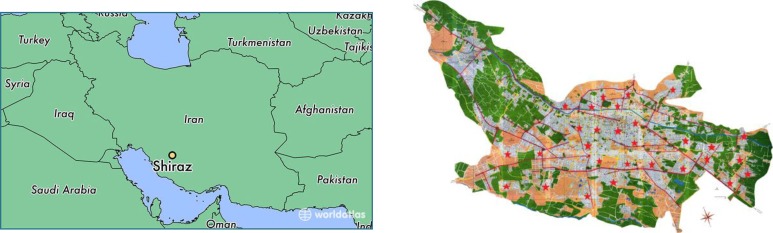
**A)** Map of Iran, showing the location of Shiraz in Fars province, Iran **B)** Map of Shiraz city with stars indicating capture areas for stray cats tested for *T. gondii* in the study

Shiraz is the sixth densely populated city in Iran and the capital of Fars province. The city’s 2014 population was approximately 1,758,000. It is situated in the mountainous region of the province with an average height of 5200 ft. above the sea level (Fig. 1 A, B).

### Ethics Statement

All experiments were arranged under the instructions and endorsement of the Institutional Animal Care Committee (IACC) of Shiraz University of Medical Sciences (approval no. 90-01-01-2817) for animal ethical and welfare standards.

The welfare of an animal includes its physical and mental state and we consider that good animal welfare implies both fitness and a sense of well-being. An animal’s welfare, whether on farm, in transit, at market or at a place of slaughter should be considered in terms of “Five Freedoms”: 1- Freedom from Hunger and Thirst, 2- Freedom from Discomfort, 3- Freedom from Pain, Injury or Disease, 4- Freedom to Express Normal Behavior, 5- Freedom from Fear and Distress.

### Sample Collection

Overall, 145 stray cats were lured alive using Sherman baited cage-traps with tinned fish, from the streets or residential areas of the city of Shiraz, Iran in 2017 and brought to the Animal Laboratory Center affiliated of the university. Thirty-five out of 145 stray cats were clinically ill. In consultation with a trained veterinarian, they had significant clinical features such as Fever, Drowsiness, Emaciation, Diarrhea, Vomiting, Jaundice, and Ocular inflammation. The remaining 110 healthy cats were released. General data on these 35 clinically ill cats including age, gender, and health status were estimated on the basis of body condition and examination of dentition (data not shown). Zoletil (Virbac, Sydney, Australia, 10 mg/kg, consist of Tiletamine and Zolazepam) were injected for anesthetic and sedative effects. Approximately 3.0 mL of blood was collected from the saphenous veins of the cats into plain sterile tubes, left to clot at room temperature for 3 h, and centrifuged afterward at 1000 g for 10 min. The separated sera were stored at −20 °C until analysis. In addition, blood samples of cats were placed into tubes containing EDTA for the analysis of *T. gondii* DNA by Nested-PCR. Amidst the 35 ill stray cats, 29 cats were positive for *T. gondii*. Amongst the cats, 21 were male and the remaining 8 were female. Subsequently, Tissue (brain, tongue, liver, spleen, ileum, kidney, diaphragm, heart, intercostal muscle) and feces samples were collected from deceased cats and examined by Nested-PCR. Formalin-ethyl acetate concentration method was applied for detection of oocysts. Tissue samples were collected from the 29 cats which were severely ill after they died instinctively in the Animal Laboratory Center under good animal welfare conditions.

### Modified Agglutination Test

We chose MAT because it is sensitive and specific for detecting *T. gondii* antibodies in many animals as compared to other serologic methods (15). Specific IgG antibodies to *T. gondii* were determined in cat sera by the MAT as described previously (16). A suspension of *T. gondii* RH strain tachyzoites purified from mouse peritoneal cavity was used as antigen. Purified tachyzoites were fixed in 6% Formalin and stored at 4 °C overnight. After fixation, the Formalin suspension was centrifuged, washed thrice in sterile PBS and resuspended in alkaline borate buffer (pH 8.7) containing 0.4% Bovine Serum Albumin (BSA/borate buffer) and 0.2% Sodium azide to a final concentration of approximately 2×10^8^/mL (antigen stock suspension), and stored at 4 °C until used. The antigen mixture for each plate was prepared by mixing 200 μL of Formalin-fixed tachyzoites, 2.5 mL of BSA/borate buffer, 35 μL of 2-Mercaptoethanol, and 50 μL of Evans blue dye solution (2 mg/mL water). Equal volumes (25 μL) of freshly prepared antigen mixture and serum dilutions were added into each well and mixed gently by repeated pipetting action. Sera from cats were diluted twofold starting from 1:20 to 1:2560 and assayed. MAT titers of 1:20 or higher were heeded as positive. Positive and negative sera, as controls, were incorporated in each plate with same dilutions as the test sera. The plate was covered with a sealing tape, incubated at room temperature and results read after 24 h. A negative result was when the base of the “U” bottom 96-well microplates (Nunc^TM^, Thermo Fisher Scientific, Waltham, Massachusetts, USA) contained a blue pellet; contrarily, a clear bottom of the well implied a positive result.

### DNA Extraction

For extraction of DNA, approximately 50 mg of the tissues were homogenized with a mortar and pestle and placed them in a clean 1.5 mL tube. Total genomic DNA was extracted from each tissue and stool sample using the AccuPrep^®^ Genomic DNA Extraction Kit, and AccuPrep^®^ Stool DNA Extraction Kit (Bioneer, Daejeon, Korea), according to manufacturer’s instructions. The quantification and quality control of the DNA extraction procedures were measured at 260 nm using a Nano spectrophotometer (NanoDrop 1000, Thermo Fisher Scientific, Waltham, Massachusetts, USA). The DNA was stored at −20 °C until used. All samples for PCR assays were prepared with aerosol-guard pipette tips to avoid contamination.

### Nested-PCR

The B_1_ gene Nested-PCR appears to be the most sensitive protocol (was able to detect 50 fg, approximately single tachyzoite) in the detection of *T. gondii* DNA and has been successful in identification of *T. gondii* DNA in tissues and fluids (17).

The external primers were 5′-GGA ACT GCA TCC GTT CAT GAG-3′ and 5′-TCT TTA AAG CGT TCG TGG TC-3′, producing an amplified product of 193 bp. The internal primers were 5′-TGC ATA GGT TGC AGT CAC TG-3′ and 5′-GGC GAC CAA TCT GCG AAT ACA CC-3′, producing an amplified product of 96 bp.

Briefly, the PCR conditions were composed of pre-denaturation at 94 °C for 5 min, then 40 cycles of denaturation at 94 °C for 30 sec, annealing at 57 °C for 45 sec, and extension at 72 °C for 1 min, followed by final extension at 72 °C for 10 min. The secondary annealing temperature was 62.5 °C. Positive sample (*T. gondii* RH strain) was used as positive control.

PCR products concomitant with 100 bp molecular marker (Fermentas, Vilnius, Lithuania) were visualized by UV after electrophoresis on 2% agarose gel using 1 × TBE buffer and stained with Ethidium bromide solution (Bio-Rad, Hercules, California, USA). All primers were synthesized by Macrogen Genomics Laboratories (Macrogen, Seoul, Korea).

### Sequencing and Phylogenetic Analysis

The PCR products were purified by using QIAquick^®^ Gel Extraction Kit (QIAGEN, Hilden, Germany) and sequenced through the sequencing service of Macrogen Genomics Laboratories (Macrogen, Seoul, Korea). Each purified DNA sample (15 ng/100 bp) was sequenced on both strands by the Sanger Dideoxy-terminal method using an ABI Prism^®^ Big Dye^TM^ Terminator Cycle Sequencing Ready Reaction Kit (3730*xl*; Applied Biosystems, Foster City, CA, USA) and 3.2 pmoles of each internal primers. The resulting sequences were submitted to BLASTn for similarity search with *T. gondii* sequences deposited in GenBank. Multiple alignments were performed with data related to *T. gondii* from Iran and other countries deposited in GenBank using BioEdit (7.2.5). A Maximum Likelihood (ML) tree was constructed using the MEGA-7 (18), and genetic distances were calculated with the Maximum Composite Likelihood method. Bootstrap analyses (using 1,000 replicates) were carried out to define the robustness of the finding. Gaps were treated as missing data.

### Statistical Analysis

The data were entered into the SPSS statistics (ver. 24, Chicago, IL, USA) and were analyzed using Mann-Whitney non-parametric test. *P*<0.05 was considered to be statistically significant. The degree of agreement was quantified by kappa index, with the following definitions: Poor agreement (k<0.20), Fair agreement (k=0.21–0.40), Moderate agreement (k = 0.41–0.60), Substantial agreement (k = 0.61–0.80) and Perfect agreement (k=0.81–1.00) (19).

## Results

Direct examination of the cats’ feces showed no oocysts. However, the results of MAT indicated that the rate of *T. gondii* infection was 82.8%. The prevalence of *T. gondii* based on gender and geographical region of the stray cats in Shiraz concluded by MAT and PCR has been displayed in Tables 1 and 2, respectively. Correspondingly, the only significant difference in MAT results possessed by the lowest infection in the cats of the central regions of the city (*P*=0.02). Supplementally, no significant kinship was discerned between the rate of infection and animals’ gender.

**Table 1:** The prevalence of *T. gondii* based on gender in stray cats of Shiraz determined by MAT and PCR

***Technique***	***MAT (positive)***		***PCR (positive)***	
***Gender***	***Number***	***Percent***	***Number***	***Percent***
Male	19	90.5	14	66.7
Female	5	62.5	6	75
Total	24	85.7	20	69

**Table 2:** The prevalence of *T. gondii* in stray cats in different regions of Shiraz determined by MAT and PCR

***Technique***		***MAT (positive)***		***PCR (positive)***	
***Region (number)***					
Center	9	5	55.5	6	66.7
North	6	6	100	4	66.7
South	2	2	100	1	50
West	4	4	100	2	50
East	8	7	87.5	7	87.5
Total	29	24	82.8	20	69

**Fig. 2:**
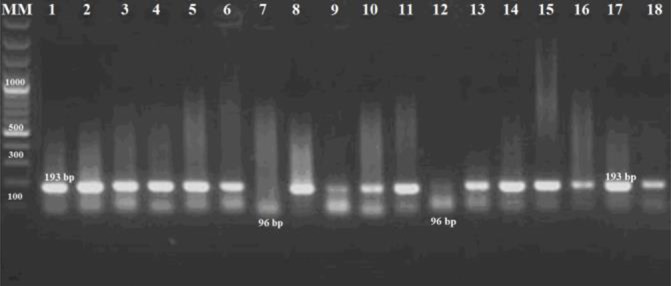
The amplicons produced from tissues in Nested-PCR based on the B_1_ gene primers. MM, 100 bp Molecular Marker (Fermentas, Vilnius, Lithuania), Positive tissue samples (lane 2–18), and reference RH strain of *T. gondii* from Tehran University of Medical Sciences (lane 1). The 193 bp amplified product belonged to the first stage of Nested-PCR, whereas the 96 bp amplified product belonged to the following stage of the test.

The results of Nested-PCR were positive in 69% of the cats. The yielded amplicons based on the B_1_ gene primers from positive samples have been presented in Fig. 2.

**Fig. 3:**
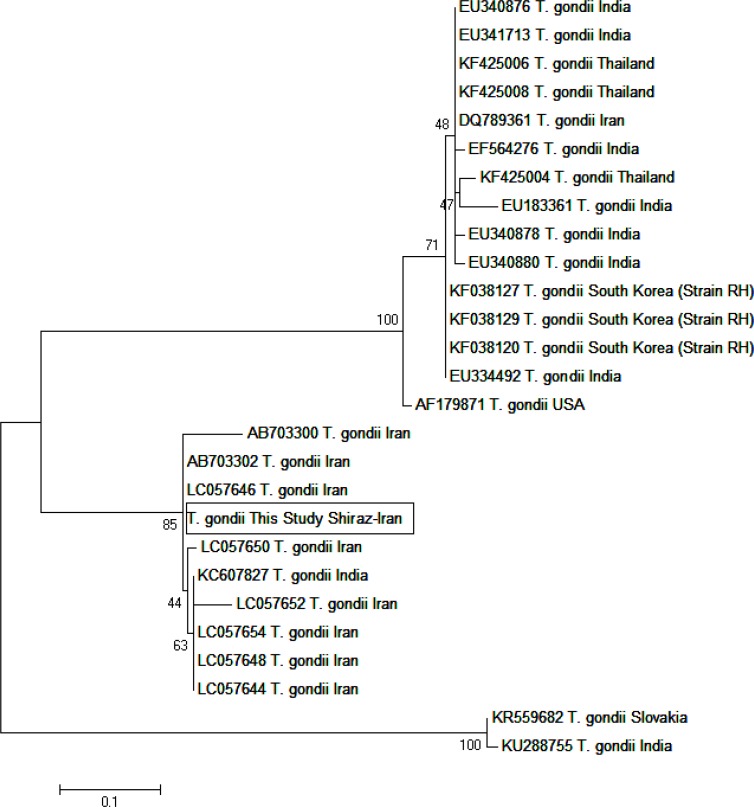
Phylogenetic relationship among various *T. gondii* to each other as inferred by Maximum Likelihood tree based on B_1_ gene. Numbers on branches are percentage bootstrap values of 1,000 replicates. All positions containing gaps and missing data were eliminated. The evolutionary distances between sequences were computed using the Maximum Composite Likelihood method and are in the units of the number of base substitutions per site. The scale bar indicates an evolutionary distance of 0.1 nucleotides per position in the sequence. The reference sequences accession numbers are inserted. Evolutionary analyses were conducted in MEGA-7.

The achieved sequences confirmed the *T. gondii* identified by the PCR analysis. The sequences of five randomly chosen of *T. gondii* infected cats showed 100% identity to the published isolate in GenBank with Accession numbers LC057646 (Khuzestan, Iran), and AB703302 (Khuzestan, Iran). Moreover, these isolates showed 99%, 98%, and 98% identity to the published isolates AF179871 (RH strain, CA, USA), AB703301 (Khuzestan, Iran), and AB703300 (Khuzestan, Iran), respectively (Fig. 3). Phylogenetic analysis of the B_1_ gene in this study revealed that our isolates were not only closely related to other isolates secluded in Iran in recent years but also had a proportionately close genetic relationship with earlier isolates from Asia and America. The results of DNA detection of *T. gondii* in stray cats’ body organs have been explicated in Table 3. The highest rate of infection was observed in diaphragm (37.9%) and intercostal muscle (34.5%), while the lowest rate was related to ileum (6.9%).

**Table 3:** DNA detection of *T. gondii* in stray cats’ body organs determined by Nested-PCR

***Technique***	***PCR (positive)***		
***Sample (number)***			
Brain	29	4	12.8
Tongue	29	6	20.7
Intercostal muscle	29	10	24.5
Heart	29	6	20.7
Liver	29	4	12.8
Spleen	29	7	24.1
Diaphragm	29	11	37.9
Kidney	29	8	27.6
Ileum	29	2	6.9
Feces	29	7	24.1
Total	29	20	69

The level of agreement between MAT and PCR in diagnosis of toxoplasmosis in the cats is shown in Table 4. The correlation between MAT with titer 1:20 and PCR was 79.2% and the correlation between MAT with titer 1:40 and PCR was 86.2% (*P*=0.02 and *P*=0.0001, respectively).

**Table 4:** Level of agreement between MAT and PCR in diagnosis of toxoplasmosis in stray cats of Shiraz, Iran

***Technique***	***PCR***		
	***Results***	***Positive***	***Negative***
MAT	*Positive*	19	5
	*Negative*	1	4

## Discussion

Insomuch cats are the only animals that expel resistant oocysts into the environment, they play the most important role in the scatter of Toxoplasmosis. Anyway, due to their special nature, oocysts are not infectious and require time for sporulation. Thus, infection is not straightforwardly passed to humans from cats. Moreover, oocyst shedding happens in short operation (12, 20). Detection of antibodies and DNA in tissues could be deliberated as a screening index of disclosure in this animal. The seroprevalence of *T. gondii* in cats varied depending on their type (stray or domestic), age, method of testing, and geography location (21).

In our study, the results of MAT revealed that the seroprevalence rate of *T. gondii* infection of cats in Shiraz was high. The rate of this infection was 86% in stray cats in the center of Iran, whereas only 4% of the cats possessed tissue cysts of *T. gondii* (22). In addition, no oocysts were detected in the cat’s stool by direct and concentration methods. Furthermore, the prevalence of *T. gondii* IgG antibodies was 40% in stray cats of northern Iran (23). This measure was also 32.1% among stray and owned cats in southeastern Iran (24). The prevalence of anti-*T. gondii* specific IgG in stray and household cats was 63% in Tehran (25). 36.71% of domestic cats were seropositive by MAT (26). In Spain, the seroprevalence of *T. gondii* in pet and stray cats were 25.5 and 36.9 % respectively (27). In the study performed on ill cats (20), 31.6 % of cats were seropositive for *T. gondii*.

In the current study, we detected *Toxoplasma* DNA in 20 out of 29 infected cats, and the parasite DNA was highly detected in diaphragm and intercostal muscle in contrast to other organs. This is in-congruent with previous studies that established the high prevalence of the parasite in infected tissues from animals and humans (28, 29). On the other side, the lowest positive rate in our study was related to ileum samples. Similar results were also being established in the previous studies (30). About, 44% of serum samples from an urban population of colony cats in Florence, Italy were positive by MAT. In addition, 16% of the fecal specimens were positive in Nested-PCR, whilst oocysts were not detected in any of the examined fecal samples (31). Antibodies to *T. gondii* by MAT was assayed and showed that 57.8% of stray cats in Beijing were seropositive. Plus, *T. gondii* oocysts were not found in feces of the cats (32). The results of a study on fecal samples of cats in Germany, Austria, and Italy showed that only 0.11% of the samples contained oocysts (33). Moreover, only 0.9% of cats’ feces in California contained *T. gondii*-like oocysts (34). Nevertheless, *T. gondii* oocysts were not established in any fecal samples in stray cats of northern Iran and only 2% of smear preparations of intestinal mucosa showed trophozoites of *T. gondii* (23). Furthermore, 24.75% of companion cats in Ahvaz, Iran had antibodies against *Toxoplasma*, but oocysts were not detected in any of the samples (35). On the other hand, *Toxoplasma* oocysts were detected in 9% of fecal samples from stray cats in northern region of Nile Delta in Egypt (36).

The results of molecular methods in the present study displayed that only two samples from ileum were positive, whilst seven fecal samples were positive. Apparently, the high rate of positive results in fecal samples might be due to the contaminated food consumed by the cats rather than shedding of oocysts.

The findings of the present study revealed that the prevalence of infection was low in the intestinal phase. Plus, the duration of *T. gondii* oocyst shedding was short. Additionally, the concurrence rate between MAT and PCR was high (79.3%). Besides, MAT was as effective as other serological tests and only one PCR-positive cat was seronegative.

The positive infection rate in stray cats of Seoul, Korea was 38.9% using PCR and ELISA (37). However, the accord between the two methods (68.1%) was lower than that of our study. On the other hand, the prevalence of *T. gondii* infection in feral cat populations in Seoul was 15.8% and 17.5% using ELISA and PCR, respectively, which were lower in contrast to stray cats in that city (37, 38). The prevalence of *T. gondii* infection among stray cats in the Gyeonggi Province, Korea was 8.0%, 16.1%, and 13.2% via LAT, ELISA, and PCR of the B_1_ gene, respectively (39).

In cat populations in North Carolina, seroprevalence of *T. gondii* was higher in feral cats than in pet cats, although the seroprevalence of Toxoplasmosis was higher in pet cats in contrast to outdoor cats, which corroborated the matter for restraining outdoor approach for pet cats (40). However, household cats in Seoul, Korea were free from infection (37).

## Conclusion

Toxoplasmosis was extensive among the stray cats of Shiraz. Since consumption of tissue cysts is an important path of infection for human Toxoplasmosis, averting assesses of the disease are suggested to be followed especially by pregnant women. Additionally, the consequences disclosed that the rate of *T. gondii* infection was 69% by PCR and 82.8% by MAT. The unanimity between MAT and PCR in diagnosis of Toxoplasmosis was also 72.4%, which was statistically meaningful.
